# Principles, Applications, and Future Evolution of Agricultural Nondestructive Testing Based on Microwaves

**DOI:** 10.3390/s25154783

**Published:** 2025-08-03

**Authors:** Ran Tao, Leijun Xu, Xue Bai, Jianfeng Chen

**Affiliations:** School of Electrical and Information Engineering, Jiangsu University, Zhenjiang 212013, China; 2222407007@stmail.ujs.edu.cn (R.T.); baixue@ujs.edu.cn (X.B.); jianfengchen@ujs.edu.cn (J.C.)

**Keywords:** agricultural nondestructive testing, microwave technology, millimeter wave measurements, intelligent detection systems

## Abstract

Agricultural nondestructive testing technology is pivotal in safeguarding food quality assurance, safety monitoring, and supply chain transparency. While conventional optical methods such as near-infrared spectroscopy and hyperspectral imaging demonstrate proficiency in surface composition analysis, their constrained penetration depth and environmental sensitivity limit effectiveness in dynamic agricultural inspections. This review highlights the transformative potential of microwave technologies, systematically examining their operational principles, current implementations, and developmental trajectories for agricultural quality control. Microwave technology leverages dielectric response mechanisms to overcome traditional limitations, such as low-frequency penetration for grain silo moisture testing and high-frequency multi-parameter analysis, enabling simultaneous detection of moisture gradients, density variations, and foreign contaminants. Established applications span moisture quantification in cereal grains, oilseed crops, and plant tissues, while emerging implementations address storage condition monitoring, mycotoxin detection, and adulteration screening. The high-frequency branch of the microwave–millimeter wave systems enhances analytical precision through molecular resonance effects and sub-millimeter spatial resolution, achieving trace-level contaminant identification. Current challenges focus on three areas: excessive absorption of low-frequency microwaves by high-moisture agricultural products, significant path loss of microwave high-frequency signals in complex environments, and the lack of a standardized dielectric database. In the future, it is essential to develop low-cost, highly sensitive, and portable systems based on solid-state microelectronics and metamaterials, and to utilize IoT and 6G communications to enable dynamic monitoring. This review not only consolidates the state-of-the-art but also identifies future innovation pathways, providing a roadmap for scalable deployment of next-generation agricultural NDT systems.

## 1. Introduction

### 1.1. Comparison of Microwave Technology with Traditional Technology and New Needs for Agricultural Testing

The quality and safety of agricultural products has always been the cornerstone of the sustainability of the global food system and public health [1,2], and the innovation of its detection technology will play a key role in guaranteeing the traceability of the supply chain, reducing the risk of foodborne diseases [3,4] and enhancing the competitiveness of international trade. According to the statistics of the Food and Agriculture Organization of the United Nations (FAO), the global economic loss due to food contamination exceeds hundreds of billions of dollars every year, which highlights the urgent need for efficient nondestructive testing technology for agricultural products [5].

**Figure 1:**
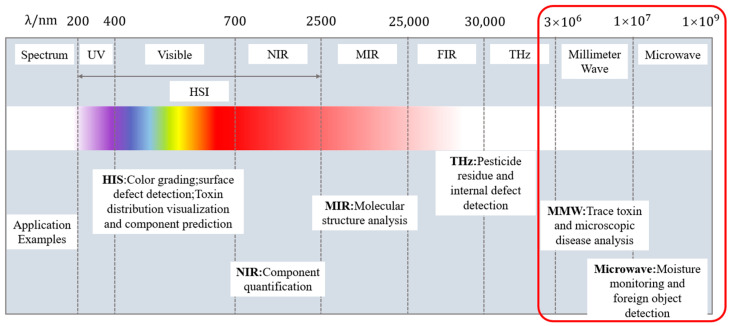
Location of microwave millimeter waves in the electromagnetic spectrum.

In recent years, techniques such as near-infrared spectroscopy (NIR) [6,7], hyperspectral imaging (HSI) [8,9] and terahertz time domain spectroscopy (THz-TDS) have been mostly used in nondestructive testing of agricultural products, but the boundaries of their application are more limited, and they are mostly used for the analysis of surface components, which need to be considered in conjunction with the specific content of agricultural product testing. As shown in Figure 1, the above techniques mainly cover the optical band of the electromagnetic spectrum. The microwave (300 MHz–30 GHz) and millimeter wave (30–300 GHz) techniques highlighted in this paper are located in the low frequency band of the electromagnetic spectrum, and due to their unique physical properties, they can penetrate the shell of agricultural products and detect the internal properties of agricultural products through the dielectric response mechanism.

The following section briefly describes the application scenarios of conventional technologies and compares them with microwave and millimeter wave technologies to clarify the different application directions of each technology and to highlight the superiority of microwave technologies.

Near-infrared (NIR) and mid-infrared (MIR) spectroscopic techniques [10,11] based on molecular vibrations with rotational energy level jumps [12,13], utilizing electromagnetic waves in the 780–2500 nm band with generalized or combined-frequency vibrations of chemical bonds [14], combined with improved clustering algorithms, have been implemented to analyze the compositions of the surface of agricultural products (e.g., apple varieties [15], tea varieties [16,17,18,19], and edible oils [20]). The Fourier transform infrared spectroscopy (FTIR) technique, based on the reconstruction of time domain spectra by the Meikle Sun interferometer [21,22], can support the analysis of trace samples [23,24,25]. However, the NIR technique is limited by the depth of penetration and is mostly used for quantitative analysis of surface composition in a laboratory setting [26,27,28]; it is dependent on the surface finish of the samples [29]; the spectral covariance of high-complexity samples [30,31] also requires complex preprocessing preprocessing steps [32]; and it usually requires long acquisition times [33,34], which restricts it from operating robustly in dynamic agricultural environments in the field [35,36]. Therefore, microwave technology with deep penetration capability is needed to complement it.

Hyperspectral imaging (HSI) is a technology that simultaneously acquires multi-band high-resolution images [37] through fusion of imaging and spectral analysis [38,39,40], and constructs three-dimensional data containing spectral–spatial information [41]. HSI is useful in monitoring crop diseases and visualizing compositional distributions [42,43] (classification of white star disease in tea [44], identification of wolfberries [45]), quantification of food components and quality analysis (e.g., soluble solids [46], degree of lipid oxidation [47,48], and detection of fruit sugars [49]), as well as in the assessment of crop storage conditions and authenticity verification (seed viability detection [50,51,52], meat freshness assessment [53,54,55], and tea species identification), etc. However, HSI often needs to [56] incorporate complex deep learning algorithms [57], and the processing of high-dimensional data leads to a lack of real-time measurement [58]; moreover, its complex optical system equipment limits deployment flexibility [59,60]. In contrast, microwave technology can achieve rapid detection of multiple parameters such as moisture and density through multi-frequency synergy and lightweight algorithms.

In addition, terahertz time domain spectroscopy (THz-TDS) is based on the interaction (absorption, scattering, and phase change) of electromagnetic waves in the 0.1–10 THz band with matter, and resolves the vibrational and rotational energy level jump characteristics of polar molecules through the dipole moment resonance effect to achieve quantitative and qualitative compositional analysis [61,62]. Although THz-TDS is excellent for crop health monitoring (e.g., tomato moisture prediction [63], apple blight diagnosis [64]), food safety screening (e.g., metal contaminant localization [65], pesticide residue identification [66]), and analysis of high-value-added agricultural commodities [67,68], the high-frequency signal attenuation, complex and expensive hardware (femtosecond lasers, cryo-detectors) [69], and the scattering effect due to the heterogeneous samples (e.g., soil–crop mixtures) make it difficult for field applicability. The effects in heterogeneous samples (e.g., soil–crop mixtures) result in limited field applicability. In contrast, microwave technology provides a cost-effective solution for grain storage detection by balancing low-frequency penetration with high-frequency resolution, combined with low-cost hardware and metamaterial enhancement.

A detailed technology comparison of the previous technology in different dimensions can be seen in Table 1. For agricultural products, nondestructive testing provides a solution; however, for agricultural scenarios, there is an urgent need to solve the difficulties of grain silo real-time monitoring, dynamic anti-interference, and field deployment [70]. This study, which contributes to the microwave and millimeter wave technology field, shows a stronger suitability [71]. The core advantage of the technology is that, based on the dielectric response characteristics, it can synchronize moisture, density, and foreign matter and other parameters of the integrated detection, significantly improving detection efficiency. The deep penetration ability of the low-frequency band breaks through the bottleneck of the existing technology that is limited to surface analysis, and it can non-invasively analyze the internal information of the material [72], while the high-frequency band can realize sub-millimeter defect identification [73]; this technology demonstrates strong environmental adaptability, and can be stably applied to dust, temperature and humidity fluctuations in complex field scenes [74]. In recent years, microwave sensors based on planar circuits—such as microstrip lines and coplanar waveguides—have advanced significantly. For example, metamaterial-enhanced coupling designs have markedly improved sensitivity, enabling the reliable identification of subtle quality changes [75]. These planar microwave sensors are compact, readily integrable, highly tailorable, and fully compatible with CMOS processes, thereby providing critical technological support for the development of low-cost, portable microwave systems for agricultural nondestructive testing [76]. The future of hardware innovation and process integration will significantly reduce the cost of the equipment and the size of the device, so as to make it more portable and potentially suitable for large-scale applications [77].

### 1.2. Objectives of the Review

This review aims to systematically analyze the principle, application progress, and future development direction of microwave and millimeter wave technology in agricultural nondestructive testing, and to provide a technical route reference for the construction of an intelligent and high-precision agricultural nondestructive testing system [79].

## 2. Core Mechanisms and Agricultural Adaptability of Microwave Technology

### 2.1. The Physical Basis of Microwave Detection

In the application of microwave technology to the nondestructive testing of agricultural products, the complex dielectric constant ($\varepsilon \;={\;\varepsilon }^{\prime }-j\varepsilon^{\prime \prime }$) of agricultural products is used as the core physical parameter, where the real part $\varepsilon^{\prime }$ reflects the energy storage capacity of the medium, and the imaginary part $\varepsilon^{\prime \prime }$ characterizes the dielectric loss factor, which together characterize the electromagnetic response properties of the material. In the low-frequency band (1–10 GHz), dielectric loss (ε″) is dominated by ionic conductivity ($\frac{\sigma }{\omega \varepsilon_{0}}$) and dipolar relaxation described by the Debye model ($\frac{(\varepsilon_{s-\varepsilon_{\infty }})\omega \tau }{1+\omega^{2}\tau^{2}}$). In contrast, high-frequency millimeter waves (>30 GHz) excite quantized molecular rotational transitions via the photon energy E = hν, producing frequency-selective dielectric fingerprints (e.g., the 24 GHz resonance peak of water and the 240 GHz characteristic absorption of AFB1) [80]. Typically, moisture content is the dominant factor governing the dielectric properties of paddy rice. As moisture increases, both the real ($\varepsilon^{\prime }$) and imaginary ($\varepsilon^{\prime \prime }$) parts of the permittivity rise, leading to stronger microwave energy absorption [81]. The energy dissipation therein is mostly characterized by the loss tangent $tan\delta \;=\;\frac{\varepsilon^{\prime \prime }}{\varepsilon^{\prime }}$, which is higher in moisture-rich agricultural products, which undoubtedly exacerbates the microwave attenuation [82,83,84]. In addition, the $\varepsilon^{\prime }$ and $\varepsilon^{\prime \prime }$ of a material determine its reflection coefficient (S11) and transmission coefficient (S21), and the electrical characteristics can also be inversely characterized by measuring these parameters [85]. Alternatively, when a microwave sensor (e.g., microstrip patch antenna, resonant cavity) is in contact with a sample, the dielectric properties of the sample change its resonant frequency, thereby resolving the dielectric constant and flow parameters of the liquid [86].

Many factors influence the dielectric properties of agricultural products; we detail them below. Firstly, water molecules are strongly polar. Free water exhibits pronounced dielectric relaxation loss at higher frequencies [87]. In peanuts, barley, walnuts, and other agricultural products, the dielectric constant rises markedly with increasing moisture content. Secondly, protein content also influences dielectric behavior [88]. Proteins contain polar groups and form hydrogen bonds with water, thereby altering the state and relaxation dynamics of bound water. The interaction between protein components in surimi seafood and microwave processing is a representative example [89]. Temperature further modifies dielectric properties by changing molecular thermal motion and the relaxation time of water. Frequency is another key factor, because both permittivity and loss factor are frequency-dependent, and the dominant loss mechanism may vary with frequency [90]. Density and packing state determine the number and arrangement of polar molecules per unit volume, affecting the overall dielectric response; these parameters must therefore be considered as potential interference factors in microwave sensing. Finally, the material’s physical structure—particle size, shape, and porosity—affects the propagation path and scattering of electromagnetic waves, and consequently the meas A Modified Formula for Calculating Dielectric Properties of Granular Agricultural Products in the Microwave Band [91].

In terms of penetration properties, microwaves can penetrate through non-conductive shells to the interior. It is worth mentioning that there is a close relationship between the microwave penetration rate and the frequency of use and the complex permittivity of the medium, which forms the theoretical basis of the detection method [92]. When microwave radiation passes through an agricultural medium, the penetration depth $\delta_{p}$ represents the distance traveled when the microwave energy is attenuated to $\frac{1}{e}$ (about 36.8%) of its surface value, and is mainly determined by the operating frequency and the dielectric loss, which can be described by a simplified equation: penetration depth $\delta_{p}\propto \frac{1}{f\sqrt{\varepsilon^{\prime }tan\delta }}$ [93]. In NDT practice, this limits the choice of operating frequencies. The essence of frequency selection lies in balancing three factors: penetration depth, spatial resolution, and molecular specificity. The microwave photon energy (E = hν, where h is Planck’s constant and ν is the photon frequency) must match the rotational energy-level spacing ∆E of a molecule to trigger a quantum transition; for example, the rotational level spacing of a water molecule ($∆E\approx 10^{-3}\mathrm{eV}$) corresponds to the millimeter wave band [94]. Low-frequency microwaves (1–10 GHz) drive ion migration and dipole relaxation through an alternating electric field (relaxation time τ≈1/(2πf)), whereas high-frequency millimeter waves (>30 GHz) possess photon energies $E>k_{B}T$ ($k_{B}$ is the Boltzmann constant) and therefore directly excite molecular rotational quantum states [95]. Lower frequencies (e.g., 1–3 GHz) deliver greater penetration—on the centimeter scale—making them well-suited for internal monitoring within grain silos. Mid-range frequencies (e.g., 5–10 GHz) provide higher spatial resolution—down to millimeters or sub-millimeters—and elevated sensitivity to grain moisture, which is ideal for near-surface analysis and compositional discrimination. Millimeter waves (30–300 GHz) align with rotational energy levels of molecules, enabling molecular-specific resonance detection, such as the identification of toxins [96,97,98]. Together, these core parameters determine the distribution of microwave signals within the product, affecting the measurement sensitivity of the reflection and transmission coefficients, thus providing a physical basis for defect identification [99].

Due to the multi-physical field coupling characteristics of the interaction between microwave and agricultural products, the density of grains also affects the overall dielectric response through the number of active molecules per unit body [100], and the temperature alters the polarization relaxation time through the molecular thermal motion; the law of synergistic variation in the dielectric spectrum with moisture–temperature provides the basis for optimizing the drying parameters in the monitoring of the drying process [101]. The dual-frequency measurement method can eliminate the density interference through the frequency difference and realize moisture detection [82]. Time domain reflection technology can also realize real-time moisture monitoring of potato starch through waveform resolution [102,103,104]. In microwave dynamic detection, the resonance effect can significantly enhance the sensitivity: when the microwave frequency matches the characteristic frequency of the target, the small change in the quality factor of the resonant cavity can also detect metallic foreign objects or pest activities [104,105,106]. It is thus possible to synchronize the inversion of moisture, density, and maturity indicators based on the transfer function of dielectric properties and physicochemical parameters [107].

Finally, selective heating properties give microwaves a unique application advantage: through dielectric differences, microwaves can target moisture in the pest to achieve inactivation while reducing the thermal impact on agricultural products [108].

In conclusion, the energy coupling between electromagnetic waves and agricultural products is the core physical basis of microwave nondestructive testing [109], and this mechanism provides nondestructive, time-sensitive technical support for agricultural nondestructive testing and lays the theoretical cornerstone for the expansion of subsequent application scenarios.

### 2.2. Band Selection and Agricultural Scenario Adaptation

The suitability of microwave technology in the nondestructive testing of agricultural products is based on the interaction between microwave technology and substances, in which the selection of frequency bands is the core element that determines the detection efficiency. Different frequency bands of microwaves have differentiated propagation characteristics and material response mechanisms, which need to be adapted according to the physical properties of agricultural products (such as water content, density, and structural complexity) and detection goals (internal defect identification, composition analysis, or dynamic monitoring).

Microwave frequencies below 1 GHz offer a unique advantage: exceptional penetration depth. This capability makes them ideal for large-scale whole-grain moisture monitoring in silos [110,111]. In soil moisture sensing, Time Domain Reflectometry (TDR) and Frequency Domain Reflectometry (FDR) sensors routinely operate at a few hundred megahertz. These signals can penetrate the soil to a usable depth, providing actionable data for precision irrigation [112,113]. Moreover, the sub-1 GHz band is relatively insensitive to density variations. This property benefits from online moisture monitoring of flowing grain and enables preliminary screening of internal moisture in thick-skinned fruits.

In low-frequency band (1–3 GHz) applications, the deep penetration property of microwaves makes them the first choice for internal inspection of granular agricultural products. Trabelsi et al. confirmed through a series of studies that electromagnetic waves in this band can penetrate the grain stacking structure and achieve simultaneous determination of moisture content and density under single-frequency conditions through dielectric spectroscopy [114], a property that can be expanded for applications in warehouse monitoring. In addition, Reimer’s team found that microwave signal perturbations caused by insect activities can be effectively captured in the 2.4 GHz band, providing a new method for nondestructive monitoring of pests in grain silos [104]. Moreover, Xie’s team further optimized the low-frequency application scenarios and developed a Doppler effect-based seed counting system, which achieves an identification accuracy of 98.3% of single/double seeds in the 1.8–2.5 GHz range [115].

The higher frequency bands (5–10 GHz and above) exhibit high sensitivity to moisture content, and Kraszewski’s classic study showed that the sensitivity of 10 GHz microwaves for detecting the distribution of moisture in the skin of fruits and vegetables was more than three times higher compared to the lower frequency bands [81]. This property has been deepened in food quality testing, where Zainal Abidin’s team found that the dielectric signature peaks at 7.43 GHz and 31.19 GHz specifically distinguish pork from other meats [116]. Xu’s latest research pushes high-frequency detection to the molecular level, with the construction of a quantitative model for aflatoxin B1 in wheat through transmission spectroscopy at 2.5–11.5 GHz (R^2^ = 0.96) [98]. Matsuo’s team broke through the bottleneck of detection in high-humidity environments by utilizing a 5.8 GHz transmission line sensor to achieve a silage moisture prediction error of <1.8% [103].

The proposal of a multi-frequency cooperative detection strategy marks the frequency band adaptation into the system optimization stage. The system developed by Lei’s team can synchronously obtain the information of shell structure strength and kernel moisture in a single measurement, avoiding the time loss of traditional step-by-step detection [117]. In addition, the Metasurface Plane Wave Antenna developed by Li’s team supports multi-frequency dielectric scanning and can be used as an extension of the technology [118]. Kumar’s review points out that the fusion of multi-frequency data can compensate for the blind spot of a single frequency band, e.g., in the assessment of seed vigor, the combination modeling of the dielectric loss factor at 2.45 GHz and phase angle at 5.8 GHz improved the prediction accuracy. Combined modeling improved prediction accuracy to 92.7% [119].

### 2.3. Advantages and Limitations

Microwave technology shows significant advantages in nondestructive testing of agricultural products. Its non-contact nature avoids the damage to samples caused by traditional destructive methods [120,121]; it is especially suitable for materials that need to maintain their physiological activity and supports the need for continuous online monitoring [122]. The technology has high sensitivity to the moisture content of grain, in the 300 MHz–1 GHz frequency band, by analyzing the microwave signal amplitude attenuation, phase shift, and resonance frequency change and other multi-dimensional parameters, combined with machine learning algorithms to achieve multi-dimensional quality assessment of moisture, density, composition, and other multi-dimensional qualities, which perform better than near-infrared and other surface technologies. The capability of low-frequency microwave penetration can detect the internal moisture content of an entire bag of material using gradient distribution visualization analysis [123]. The development of portable devices (miniaturized antennas, low-power circuits) in the last five years has further reduced the cost of field deployment and enhanced the potential for technology penetration [124,125,126].

However, the technique faces some limitations. The detection signal is susceptible to interference from ambient temperature, humidity, electromagnetic noise, and the sample’s own physical properties (e.g., shape, surface roughness), and needs to be compensated for errors through dynamic calibration. Dynamic changes in the dielectric constant due to the growth period or storage conditions may also lead to model prediction bias [127], requiring the establishment of multi-scenario calibration databases (e.g., the microwave imaging repository at the University of Manitoba) [128,129]. Another core challenge lies in the trade-off between penetration depth and resolution: at too high a frequency, the effect of inter-particle voids is not negligible according to the equivalent medium theory; at too low a frequency, the coupling efficiency between the electromagnetic wave and the material particles decreases, leading to a reduction in sensitivity [78,130].

In conclusion, with the advantages of nondestructive, high penetrability, high sensitivity to moisture, and fast response, microwave technology has become one of the mainstream methods for nondestructive testing of agricultural product storage [131]. However, its wide application still needs to overcome the limitations of environmental interference, sample dependence, the contradiction between penetration depth and resolution, hardware cost, and data processing complexity.

## 3. Extended Application Scenarios of Microwave Technology

### 3.1. Typical Application Scenarios: Moisture Content Detection

#### 3.1.1. Cereal Crop Moisture Testing

Wheat: As a major global food crop, wheat moisture detection technology presents a mature solution with multi-frequency band synergy. For example, Bai et al. achieved moisture prediction accuracy significantly better than traditional methods through double-mixed-frequency microwave transmission (2.5–11.5 GHz) combined with support vector regression (SVR) modeling [132]. Kuang et al. further utilized the L/S-band frequency modulated continuous wave system to establish a full-frequency SVR model, which verified the superiority of multi-frequency data fusion [107]. Li et al. developed a dielectric hypersurface lens antenna system (23.6–24 GHz) to achieve density-independent high-precision detection through an error calibration algorithm [133].

Corn and Soybean: Zhang et al. developed a multi-frequency scanning combined with a deep neural network online sensing system for real-time monitoring of the corn drying process [72]. Nelson et al. proposed a microwave density-independent technique for the simultaneous detection of moisture in corn, soybean, and other flowable grains through attenuation and phase-shift measurements [134]. Li et al., based on the density-independent square of the traveling-wave standing-wave method, further verified the suitability of microwave technology for a wide range of grains [135].

#### 3.1.2. Oil-Bearing Crop

Peanuts: Trabelsi and Nelson achieved simultaneous determination of moisture and bulk weight of shelled peanuts by complex dielectric constant analysis [136]. Ma et al.’s team constructed a fully connected deep neural network model based on microwave scattering parameters, which significantly improved the detection efficiency [137]. For peanut shell particles, Trabelsi’s team also proposed a multi-band (5–15 GHz) density-independent calibration function, which can be used to predict moisture and bulk weight in milliseconds [138].

Unshelled tea seeds: A microwave detection system based on multi-frequency scanning (2.00–10.00 GHz), combining artificial neural network (ANN) modeling and raw moisture calibration function, was developed by Zhou et al. to achieve high-precision prediction of the internal moisture of tea seeds in the unshelled state [139].

#### 3.1.3. Other Materials

Tea leaves: Wu et al. developed a moisture prediction model for the withering process using a fusion strategy of 2–10 GHz multi-frequency signal optimization and machine learning, providing a new paradigm for quality control in tea processing [140].

Plant leaves: Yan et al. designed a 3.4 GHz split-ring resonator sensor to monitor leaf moisture through frequency shifts for non-invasive real-time detection [141]. Menzel et al. successfully tracked the moisture dynamics of tomato and tobacco leaves based on the linear relationship between the frequency shift of the center of the microwave resonator cavity and the fresh weight of the plant [100,142].

Silage: For high-moisture forage crops, Matsuo et al. proposed a phase-amplitude ratio method (3.6 GHz) based on a microstrip transmission line to solve the problem of nondestructive inspection of packaging films [82].

The key metrics of the case are shown in Table 2, which indicates that microwave technology has achieved the full chain of moisture detection coverage from stored grains to field crops through frequency band adaptation, algorithm optimization, and multi-physical quantity synergy.

### 3.2. Emerging Application Directions

#### 3.2.1. Agricultural Storage and Processing Monitoring

As shown in Table 3, taking biofilm monitoring in dairy pipelines as an example, Bhattacharya’s team found through numerical simulation of electromagnetic fields that a biofilm with a thickness of 1 μm can cause significant changes in reflection coefficients in the 1.52 GHz band. This sensitivity is more than five times that of conventional optical methods, providing a new paradigm for continuous online monitoring [143]. Similar principles can be extended to the identification of stored grains: Wang et al. established a rapid nondestructive detection method for new/old rice grain adulteration utilizing broadband microwave transmission (8–12 GHz). By analyzing the transmission coefficient (S21) spectral features and employing a Support Vector Machine (SVM) classifier, the method achieved high identification accuracy, significantly outperforming traditional sensory evaluation in both speed and objectivity [144].

#### 3.2.2. Mycotoxin Detection

Microwave and millimeter wave technologies provide innovative solutions for on-site screening of mycotoxins due to their high sensitivity to the dielectric properties of materials. The underlying principle is that polar molecules (e.g., the carbonyl groups of aflatoxins) exhibit the Stark effect ∆E = −μE in an intense microwave electric field, where μ is the dipole moment. This interaction splits rotational energy levels and enhances resonant absorption [145]. Aflatoxin B1 (AFB1) contains a strongly polar lactone ring (C–O–C=O) and furan ring (C_4_H_4_O) with a dipole moment μ≈3.5Debye. The reduced rotational barrier of the molecule shifts its characteristic relaxation peak to 31 GHz ($\varepsilon_{max}^{\prime \prime }\propto \mu^{2}/T$), clearly distinguishing it from the broad absorption spectrum of the cereal matrix [146]. In AFB1-contaminated wheat, the $\varepsilon^{\prime \prime }$ peak at 31 GHz rises to ≈0.85, which is significantly higher than that of uncontaminated samples (≈0.15). A secondary peak at 11.5 GHz ($∆\varepsilon^{\prime \prime }\;=\;0.32$) provides an additional discrimination dimension, forming a dual-band dielectric fingerprint [78].

In recent years, researchers have built an integrated monitoring system for the grain storage environment by combining microwave sensing technology with thermodynamic parameter analysis through a multimodal data fusion strategy. For example, to address the bottleneck of highly toxic aflatoxin B1 (AFB1) detection, Xu et al. developed a miniaturized microwave sensing system (2.5–11.5 GHz), which was optimized by the transmission exponential (S21 phase-to-amplitude ratio) and the Support Vector Machine regression (SVR) model, and the root-mean-square error (RMSEP) of the quantitative detection of AFB1 was reduced to 2.8 μg/kg [98]. To further break through the challenge of synergistic optimization of qualitative–quantitative detection, Deng’s team proposed a multi-task deep learning framework to synchronously invert wheat mildew grade and AFB1 content by using the frequency domain features of microwave transmission coefficients, which provides a full-dimensional technological support for the quality grading of warehouse monitoring [123]. It is also worth noting that, in high-moisture samples (>15%), the strong frequency-dispersive absorption of free water ($\varepsilon_{water}^{\prime \prime }\propto f\cdot c_{\omega }$, where $c_{\omega }$ is the grain moisture content) across 1–20 GHz masks the toxin signal. Therefore, pre-drying or a dual-frequency differential method is required to suppress the water-induced background noise [147]. In the field of plant pathogen detection, microwave technology has demonstrated the ability to specifically identify fungal types through the analysis of dielectric fingerprints. Hussein et al. found, using open coaxial probe technology, that soil-borne fungi have a higher value of dielectric loss factor ($\varepsilon^{\prime \prime }$ ≈ 0.15) in the low-frequency band (<5 GHz) compared to air-borne fungi (e.g., powdery mildew, $\varepsilon^{\prime \prime }$ ≈ 0.03) by two to three orders of magnitude, which is a significant improvement in the value of the loss tangent. This difference provides a physical basis for rapid typing and targeted control of pathogens in the field [148]. Expanding to the safety monitoring of edible oils, the combination of microwave spectral data and deep neural networks further breaks through the sensitivity limit of trace contaminant detection. Deng et al. achieved ppb-level detection of lead residues in edible oils (LOD = 0.5 ppb) through broadband dielectric spectral (1–20 GHz) feature extraction and convolutional neural network (CNN) modeling, and the generalization of their model for heterogeneous oil samples error is less than 8% [123]. These advances have not only verified the technical advantages of microwave technology in toxin detection, but also laid a methodological foundation for the construction of a whole chain food safety prevention and control system from field to table.

#### 3.2.3. Food Adulteration Identification

Compared with optical technology that relies on surface features, microwave technology provides a unique solution for accurate identification of heterogeneous components in powder products by virtue of its deep penetration capability and dielectric response characteristics. Taking wheat flour adulteration detection as an example, the microwave detection system (2.5–11.5 GHz) independently developed by Xu’s team constructs an XGBoost bimodal detection model for talcum powder adulteration through wide-band dielectric spectrum acquisition, combined with adaptive feature selection algorithms (e.g., improved ant colony optimization) and population intelligent optimization strategies. The model realized the classification discrimination and quantitative prediction of the adulteration ratio through the nonlinear mapping of frequency domain dielectric loss factor (ε″) and phase offset, which was significantly better than that of the traditional chemical analysis method, and the detection time was shortened to 30 s/sample [149].

**Table 3:** Summary of representative cases for emerging applications.

Applications	System Schematic	Measurement Technique	Frequency	Signal Processing Methods	Ref.
Milk biofilm detection	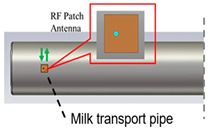	Reflection coefficient (S11) and resonance frequency shift analysis	1.52 GHz	Frequency offsets and amplitude changes	[142]
Storage grain identification	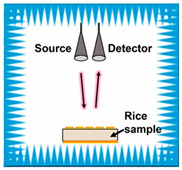	Reflection spectrum measurement (free-space method)	7.11–7.52 GHz	Full-wave simulation and measurement results analysis; multi-reflection interference theory	[144]
Wheat aflatoxin B1 (AFB1) quantification	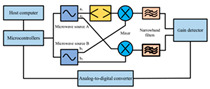	Dual-heterodyne mixing technology, broadband free-space transmission	2.5–11.5 GHz	Least squares filtering preprocessing, bootstrap soft shrinkage (BOSS) algorithm for feature optimization, and Support Vector Machine (SVM) ion (R^2^) = 0.97	[98]
AFB1 and mold co-detection	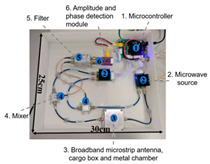	A miniaturized microwave detection device with a double heterodyne mixing structure	4.5–11.5 GHz	A multi-task CNN model for feature self-learning and model calibration	[123]
Phytopathogenic fungi differentiation	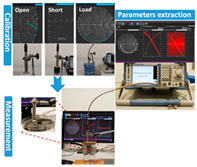	Open-ended coaxial probe technique	200 MHz–13.6 GHz	The DAK 3.5 software extracts the dielectric parameters of the material by measuring the complex reflection coefficient at the probe end	[146]
Lead (Pb) residue in edible oil		Employing the penetrating, absorbing, and reflecting properties of microwaves to detect and analyze sample properties	300 MHz–300 GHz	Convolutional neural networks (CNNs), residual neural networks, and attention mechanisms	[147]
Talc adulteration in wheat flour	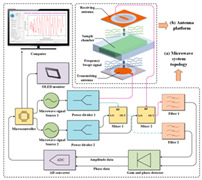	Amplitude attenuation and phase changes	2.5–11.5 GHz	Feature selection strategies (such as BOSS, CARS, and MEF-LASSO) are employed to reduce data dimensions and extract key features	[148]
Animal-derived biological tissues (beef, pork, chicken, liver, and skin)	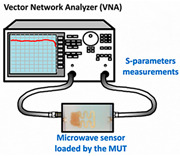	Improved ring resonator (CPW-fed, meander-miniaturized), ex vivo biological tissue capacitive perturbation measurement	0.5–5 GHz	Measure resonance shift and Q-factor change; fit S-parameters via EM simulation to extract complex permittivity	[150]

#### 3.2.4. Biometric Identification

Based on the resonant dielectric characterization mechanism, microwave sensing technology extends its application frontier to in situ tissue biometrics by resolving dielectric “fingerprints” linked to macromolecular composition. The miniaturized CPW-fed meander resonator (1 GHz) developed by Tilii et al. achieves high-sensitivity extraction of tissue-specific complex permittivity ($\varepsilon^{\prime }$ and $\varepsilon^{\prime \prime }$) through gap-coupled partial overlay, effectively capturing polarization differences in protein-bound water and cellular structures. For instance, ex vivo measurements revealed distinct permittivity ranges between muscular (e.g., beef $\varepsilon^{\prime }$ = 52.9, $\varepsilon^{\prime \prime }$ = 15.4) and parenchymal tissues (e.g., pork liver $\varepsilon^{\prime }$ = 45, $\varepsilon^{\prime \prime }$ = 14.2), correlating with histological variations in collagen density and hydration states (validation error < 5% vs. coaxial probe). This approach offers real-time, non-ionizing tissue discrimination, surpassing invasive DNA-based methods in operational simplicity for clinical diagnostics [123].

The above progress indicates that microwave agricultural detection technology is rapidly advancing from laboratory research to industrialized applications. With the deep combination of miniaturized sensors and intelligent algorithms, the technology is expected to achieve a wider penetration in the scenarios of agricultural product quality tracing, intelligent monitoring of processing, etc., and provide core technical support for the construction of an intelligent agricultural inspection system.

### 3.3. The Forward-Looking Potential of Millimeter Wave Technology

As a high-frequency extension of microwaves, the quantization energy of millimeter wave technology (30–300 GHz) matches the rotational energy level jumps of molecules, and is able to interact resonantly with water molecules, proteins, fats, and toxins in grains to stimulate changes in their rotational energy levels. Such interactions result in significant amplitude attenuation and phase changes in the millimeter wave signal as it penetrates the grain, and these changes are closely related to the compositional content and structural state of the grain. Therefore, by accurately measuring the transmission characteristics of millimeter waves, rapid and nondestructive detection of the internal qualities of grains, such as moisture content and quantitative analysis of harmful substances such as aflatoxin, can be realized.

For example, a millimeter wave system based on graphene–metal composite metamaterials achieved trace detection of aflatoxin B1 in the 120 GHz band with a sensitivity enhancement of 20 dB compared with the conventional optical method [74]. Similarly, Xu et al. achieved accurate quantitative analysis of aflatoxin B1 in peanuts by the localization of molecular characteristic absorption peaks in the 240 GHz band combined with principal component analysis and a support vector regression model [78]. All these studies show that millimeter wave technology can break through the sensitivity bottleneck of optical methods in the detection of toxins at low concentrations and provide efficient solutions for the safety of agricultural products.

In addition, the maturity and scalability of the CMOS process enable it to adapt to the high-frequency requirements of the millimeter wave band and support large-scale mass production, which provides a technological basis for the widespread application of millimeter wave grain quality sensing chips [151]. By adopting the CMOS process, it can not only realize the miniaturization and high performance of grain quality instruments, but also promote their popularity in the agricultural field, help the process of agricultural modernization, and provide a stronger technical guarantee for food security.

For example, Liu et al. designed a 5 GHz and 240 GHz dual-band synergistic system, in which the low-frequency band can globally monitor the moisture distribution of grains, while the high-frequency band can locate the local toxins through molecular resonance, thus forming a macro–micro multi-dimensional detection framework, which significantly improves the detection efficiency [152].

## 4. Challenges and Future Perspectives

### 4.1. Current Technology: Three Technical Bottlenecks

**Figure 2:**
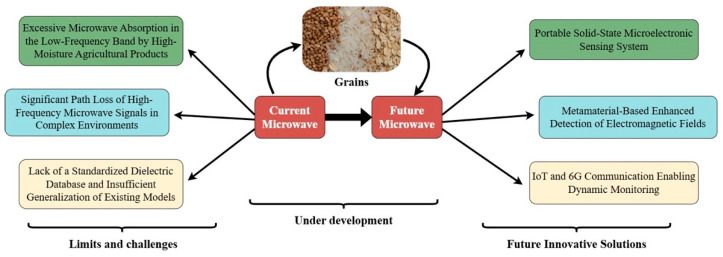
Microwave NDT in agriculture: challenges and roadmap for future evolution.

Although microwave technology shows unique advantages in nondestructive testing of agricultural products, its large-scale application still faces three major technical bottlenecks, as shown in Figure 2.

Firstly, the absorption effect of high-moisture agricultural products on specific frequency bands of microwaves interferes with the measurement accuracy. High-moisture grains (such as corn, fruits, and vegetables) in the microwave low-frequency band (1–10 GHz) on electromagnetic wave absorption are significantly enhanced, resulting in a weak reflection signal, making it difficult to accurately measure the local moisture distribution through the reflection method or transmission method. For example, the dynamic moisture measurement of corn kernels with bracts, due to signal absorption, leads to increased error [153]. In addition, Jones’ team noted that the dielectric loss factor ($\varepsilon^{\prime \prime }$) of grains with >20% moisture content rises sharply, reducing the depth of penetration and invalidating transmission-based detection [126]. Finally, research by Min and colleagues also demonstrated that moisture-induced signal attenuation can mask the identification of target features such as mold [154].

Secondly, millimeter wave high-frequency signals in microwaves have significant path loss in complex environments. Although the millimeter wave frequency band can improve the resolution, in the crop canopy and other complex environments, the path loss is significant. Path loss of millimeter waves in crop canopies follows the Friis-modified formula $L_{p}\;=\;\frac{{\left(4\pi fd\right)}^{2}}{c^{2}}\cdot \sigma_{scatter}$, where the scattering cross-section is $\sigma_{scatter}\propto r^{3}$ (r = cellulose particle radius). This explains the 25 dB loss at 60 GHz in maize fields, arising from stem scattering with r≈0.5 mm [155]. For high-moisture grain (>20%), microwave attenuation obeys the Beer–Lambert law: $\frac{I}{I_{0}}\;=\;e^{-\alpha d}$, with attenuation coefficient $\alpha \;=\;\frac{2\pi f}{c}\sqrt{\varepsilon^{\prime }}tan\delta$ reaching $8.2\;m^{-1}$ at 2.45 GHz ($tan\delta \propto c_{\omega }$), yielding a penetration depth $\delta_{p}<5\;cm$ [156]. As mentioned earlier, Xu et al.’s team validated aflatoxin detection in peanuts and found that the 40 GHz signal had a 42% signal-to-noise ratio degradation due to particle scattering and relied on complex compensation algorithms to calibrate the data [78]. At the same time, Pattanayak further noted that cellulose structures in crop residues can enhance millimeter wave scattering and exacerbate signal distortion [157]. Dielectric variability arises from variety-dependent differences in hydrogen-bond networks; for example, the increased β-sheet content in high-gluten wheat gliadin strengthens dipolar orientational polarization [158].

Finally, the microwave method is relatively new, and the dielectric properties of agricultural products are affected by the coupling of multiple factors such as variety, temperature, and density, resulting in a lack of standardized dielectric databases and a lack of generalizability of the existing models. Zhong’s team emphasized that the traditional dielectric model has an error of 12% for wheat with varying gluten content, and the Modified Model for Agricultural Productivity and Moisture Measurement (MGAPEM) is still limited to a specific range of densities [91]. Jones also pointed out that existing commercial moisture meters need to be individually calibrated for different grains (e.g., soybean vs. chickpea) because of the 3–5 times difference in dielectric constants (at 10 GHz), further highlighting the lack of a standardized database for multiple scenarios as a bottleneck [125].

### 4.2. Innovative Solutions: Future Evolution Paths

With the evolution of agricultural inspection needs to intelligence and high precision, the future development of micro-technology needs to break through single-modal limitations and build a multi-dimensional synergistic inspection system through the integration of cross-field technologies. Therefore, in view of the existing bottlenecks in microwave nondestructive testing technology for agricultural products, the future evolution path should focus on the following directions:

#### 4.2.1. Portable Sensing System Based on Solid-State Microelectronics

At present, there are relatively few studies in the field of solid-state electronics applied to grain quality inspection at home and abroad, and most of them still rely on general-purpose measuring instruments such as vector network analyzers [159,160], which makes it difficult to realize truly portable inspection. In grain quality nondestructive testing equipment, the performance of the signal source [161,162,163] and detector [164], as the core components, directly determines the accuracy and reliability of the detection results. However, if the CMOS process is used to achieve a high degree of integration of the microwave detection chip, it will directly solve the problem of equipment portability and detection sensitivity.

In addition, since the selection of semiconductor technologies—CMOS, SiGe BiCMOS, and GaN—in the field of agricultural microwave sensing requires a comprehensive trade-off among power consumption, cost, performance, and portability, we will next delve deeply into these factors and reveal the reasons behind our prioritization of CMOS technology.

CMOS (complementary metal–oxide–semiconductor) stands out for portable instrumentation because it delivers very-large-scale integration, the lowest cost, and modest power draw [151,165,166]. Its mature design ecosystem makes it ideal for battery-powered handheld moisture meters or densely integrated sensor nodes [167]. Nevertheless, CMOS is generally limited to applications below about 100 GHz [168], and its high-frequency figures of merit ($F_{T}/F_{MAX}$) and output power are comparatively modest [152].

SiGe BiCMOS technology merges the digital integration capability of CMOS with the superior high-frequency performance, high linearity, and low phase noise of SiGe HBTs, thereby delivering output power levels that surpass those of pure CMOS while achieving $F_{T}/F_{MAX}$ figures that already reach 150/200 GHz in production and 350 GHz in research [169,170]. Operating effectively in the multi-gigahertz and multi-gigabit-per-second regimes, SiGe BiCMOS is well-suited for both wireless and wireline circuits and networks [171]. Its balanced energy efficiency, which is better than that of III–V semiconductors, makes it particularly attractive for millimeter wave and high-performance applications such as high-resolution radar and precision spectroscopic analysis, although its process complexity and cost remain higher than those of CMOS [172].

GaN and other III–V compound semiconductors such as InP deliver extremely high output power, power density, efficiency, and breakdown voltage, together with outstanding high-frequency performance [173]. GaN devices can operate up to roughly 1000 GHz and deliver kilowatts of power, far surpassing both Si and SiC. However, GaN remains the most expensive option and offers lower integration density, making complex system-on-chip solutions difficult to realize. Its process maturity and ecosystem also lag behind those of silicon-based technologies [174,175]. Consequently, GaN is mainly reserved for high-power transmission tasks, such as penetrating very thick materials or enabling long-range detection, and is seldom adopted in portable agricultural equipment.

In summary, for the mainstream portable, low-cost, mid-/low-frequency or millimeter wave frontend sensing systems envisioned in this paper, CMOS technology—thanks to its unmatched level of integration, lowest cost, and lowest power consumption—emerges as the most feasible and promising choice.

#### 4.2.2. Metamaterial-Based Electromagnetic Field Enhancement Detection

The rise of metamaterial technology in recent years has provided new ideas for grain quality testing. According to the Maxwell–Garnett effective-medium theory, the effective permittivity is $\varepsilon_{eff}\;=\;[1+\frac{3\phi (\varepsilon_{d}-\varepsilon_{m})}{\varepsilon_{d}+2\varepsilon_{m}-\phi (\varepsilon_{d}-\varepsilon_{m})}]$, and the local field-enhancement factor is $\beta \left(\omega \right)\;=\;\left|\frac{3\varepsilon_{m}}{2\varepsilon_{m}+\varepsilon_{d}}\right|$, where $\varepsilon_{m}$ is the permittivity of the grain matrix and $\varepsilon_{d}$ is the permittivity of the other material [176]. In addition, metamaterials use nanoscale surface plasmon resonance periodic structures with strong absorption and modulation of electromagnetic waves. This property can be utilized to improve the sensitivity of grain quality detection [74]. In the field of microwave-based nondestructive testing of agricultural products, application-specific metamaterial structures can be selected for different scenarios. For moisture content determination in grains, an apolarization-insensitive wheel-shaped resonator exhibits ultra-high water absorption and adapts well to irregularly shaped kernels, making it suitable for moisture measurement in nuts such as almonds [177]. For grain quality assessment, a cross-resonator array detects shifts in resonant frequency to identify food adulteration or varietal differences [144]. In addition, Tian et al. used a metamaterial lens to modulate the wavefront phase, combined with an adaptive source localization algorithm to solve the resolution limitation of conventional microwave imaging. Experiments show that the detection size of insect defects inside the grain is reduced to 0.5 mm (three times higher than conventional methods) [178]. In pH measurement of aqueous solutions or dielectric characterization of solid materials, metamaterial unit cells formed by coupled open square-ring resonators exploit negative magnetic permeability (MNG) supported by transmission-line and stopband-shift techniques, enabling high-precision sensing [179]. Moreover, a compact, transmission-line-based absorber that incorporates negative permittivity elements significantly enhances broadband sensitivity and suits diverse agricultural environments [180]. Finally, Chen’s team mimics the structure of plant microtubules to design gradient dielectric constant materials, realizing the directional aggregation of electromagnetic waves to increase the depth of penetration of microwaves inside the grain by 40%, which effectively improves the detection ability of deep contaminants [181].

These examples illustrate that metamaterial architectures can enhance field localization and resonant response. Nevertheless, practical deployment must account for both the physical state of the agricultural product and environmental factors. Future integration with intelligent algorithms promises to deliver high-sensitivity detection [182].

#### 4.2.3. IoT and 6G Communications-Enabled Dynamic Monitoring

The popularization of the Internet of Things (IoT) in agriculture provides a physical vehicle for multi-technology integration. Drones equipped with agricultural sensors can acquire environmental parameters (temperature, humidity, and soil pH) in real time, combined with aerial images from drones to collect pest and disease data [183]. The development of room temperature detectors for 6G IoT applications can focus on solving the problem of high signal loss in air, and their high sensitivity characteristics can meet the needs of environmental monitoring, 6G signal reception, and other scenarios [184]. As for the 6G-IoT-based smart irrigation system constructed by Sitharthan et al., its network layer design draws on millimeter wave beamforming technology, which can reduce signal attenuation in the complex environment of farmland through directional transmission [185].

In remote, low-power agricultural scenarios, the optimal data-transmission protocol is Low-Power Wide-Area Network (LPWAN) technology, supported by lightweight data-compression algorithms to guarantee efficient and reliable delivery. The entire solution will ultimately be integrated into the 6G-IoT smart irrigation framework proposed by Sitharthan [186]. Prediction errors caused by moisture, shape variability, and dielectric heterogeneity can be overcome through online adaptive calibration. A transfer-learning-based convolutional neural network (CNN) continuously fuses multi-band dielectric spectra—$∆\varepsilon_{f1-f2}^{\prime },\;∆\varepsilon_{f1-f2}^{\prime \prime }$—with temperature and humidity sensor data in real time, dynamically adjusting the weight parameters $\varepsilon \;=\;f(T,c_{\omega },variety)$ to minimize field errors [187].

For protocol selection, LPWAN is the field-ready choice because of its ultra-long coverage and extremely low power consumption. LoRa/LoRaWAN is ideal for periodic sensor uploads, such as soil moisture and temperature. Its spread-spectrum modulation delivers kilometers of range in rural areas while drawing minimal current, making it suitable for moderate data rates [188,189,190]. NB-IoT and LTE-M, based on cellular infrastructure, offer broader coverage and lower latency with higher data rates, serving real-time applications like pest alerts [191]. Looking ahead, 6G’s massive Machine-Type Communication (mMTC) will evolve these technologies further. Reconfigurable intelligent surfaces (RISs) will dynamically optimize beamforming to mitigate the high path loss of millimeter waves in farmland, effectively relaying signals to expand coverage [192,193].

On the compression side, strict bandwidth and energy budgets demand lightweight techniques that maximize efficiency. The preferred strategy is on-node feature extraction, transmitting only critical features instead of raw voluminous data. This “intelligent compression” slashes both data volume and energy draw [194]. For spectral data, lightweight lossless or near-lossless algorithms—such as differential coding followed by Huffman coding—remove redundancy with low computational cost, fitting resource-constrained nodes [195]. For imaging data, predictive schemes like JPEG-LS preserve accuracy while saving bandwidth. When a controlled loss of fidelity is acceptable, lossy methods such as WebP provide higher compression ratios, provided the trade-off between algorithmic complexity and information loss is carefully managed [196].

### 4.3. Discussion on Limitations and Future Perspectives

Beyond the fundamental hurdles of signal attenuation in high-moisture products, millimeter wave path loss, and the absence of standardized dielectric databases, broader challenges impede practical deployment: limited model generalizability across crop varieties and environmental conditions; rising system complexity and costs from multi-technology integration; insufficient standardization for regulatory compliance; vulnerabilities in field robustness (temperature, humidity, and vibration); stringent energy constraints for remote operations; and edge-computing bottlenecks for real-time data processing.

Future advancements require focused efforts on multimodal sensor fusion (e.g., microwave–optical synergy) for comprehensive analysis [197,198]; AI-optimized hardware/software co-design to enhance adaptability; dynamically tunable and low-cost metamaterials; open-access dielectric repositories with cloud-based calibration; dedicated CMOS-integrated circuits for miniaturized low-power systems; accelerated environmental stress testing; rigorous cost–benefit assessments of deployment models; and expansion into novel applications like root-zone hydration mapping and physiological stress early-warning systems. Overcoming these multifaceted barriers is essential to transition microwave NDT from laboratory validation to scalable agricultural intelligence platforms.

## 5. Conclusions

Microwave technologies have shown significant technical advantages and wide application prospects in the field of agricultural nondestructive testing (NDT) by virtue of their nondestructive detection characteristics and deep penetration ability. Through the penetration of the low-frequency band (300 MHz–4 GHz) and the molecular resonance effect of millimeter waves (30–300 GHz), the complementary detection framework of “macro-screening–micro-diagnosis” has been constructed by the two technologies, which can be realized from the monitoring of moisture to the identification of trace toxins in stored grains. In terms of hardware innovation, CMOS multimode oscillators significantly reduce device size and power consumption. When coupled with metamaterial-enhanced techniques, microwave technology gains the capability for high-precision, low-cost, nondestructive testing. This combination improves signal stability in complex environments and provides robust hardware support for real-time, on-site detection. The ability to detect synergistic multi-dimensional physical quantities (e.g., moisture, density, and foreign matter) allows it to demonstrate unique advantages over traditional optical methods in dynamic agricultural environments and complex sample analysis.

Despite the outstanding advantages of microwave technology, its large-scale application still faces three major technical bottlenecks: excessive absorption of microwaves in specific frequency bands by high-moisture agricultural products, significant path loss of millimeter wave high-frequency signals in complex environments, and the lack of a standardized dielectric database. Future breakthroughs will focus on portable sensing systems based on solid-state microelectronics to enhance field adaptability; electromagnetic field-enhanced detection technologies utilizing metamaterials to improve sensitivity and penetration depth; and the combination of Internet of Things (IoT) and 6G communication-enabled dynamic monitoring networks to realize the whole process of intelligent management from field to storage. Through the in-depth integration of interdisciplinary technologies and full scene coverage, microwave technology not only builds a highly reliable technology system for the quality and safety of agricultural products and supply chain management, but also provides both prospective and practical solutions for the sustainable development of global food security, and will promote agricultural testing towards a new paradigm of intelligence and high precision.

## Figures and Tables

**Table 1:** Comparison table of nondestructive testing techniques.

Technical Dimension	NIR and FTIR	HIS	THz-TDS	Microwave	Millimeter Wave
Frequency range [3]	700–2500 nm	400–2500 nm	0.1–10 THz	300 MHz–3000 GHz	30–300 GHz
	2.5–25 μm				
Major applications [66]	Surface composition analysis	Visualization of toxin distribution	Pesticide residue detection	Moisture detection	Trace toxin detection
Detecting sensitivity [66]	Tolerance ± 0.3–2.1 μg/kg	Tolerance 6.65 mg/k	Sensitivity 94.51–100%	Tolerance < 0.5%	Limit of detection 0.1 μg/kg
Scope of application [23]	Laboratory precision composition analysis	Imaging of toxin distribution in surface homogeneous samples	High-precision molecular fingerprinting in the laboratory	Real-time field testing	High value-added scenarios
Equipment cost [7,63]	Medium (tungsten halogen lamp + silicon-based detector)	High (high-precision spectrometer + imaging system)	Very high (cryogenic detector + precision optics)	Low (portable antenna + CMOS chip)	Medium (CMOS integrated design)
Field suitability [3]	Requires shading and is susceptible to dust disturbance	Dependent on a stable light source, with weak outdoor adaptability	Requires a dry nitrogen environment, complicated pre-treatment	Strong adaptability (stable operation in rain and fog)	Highly resistant to interference (supports real-time detection of dynamic environments)
Penetration [78]	Low (surface micron level)	Low (surface detection)	Medium (millimeters, suppressed by moisture)	High (centimeters, penetrating non-metallic materials)	High (millimeters to support package integrity testing)
Data dimensions and processing efficiency [49]	One-dimensional spectra with simple processing	Three-dimensional data cube, requiring dimensionality reduction	Time domain spectroscopy + imaging with terabytes of data	1D/2D imaging with real-time processing	Multi-dimensional signal fusion with support for edge computing

**Table 2:** Microwave-based moisture detection in agricultural products: representative applications.

Material	System	Detection Principle	Key Metrics	Ref.
Wheat	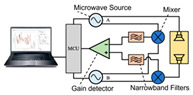	Combining Competitive Adaptive Re-weighted Sampling (CARS), Genetic Algorithm (GA), and Support Vector Regression (SVR) feature selection and modeling.	R^2^ = 0.976	[129]
Wheat	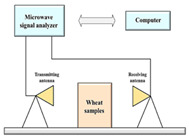	Multi-band (L/S) dielectric response; Partial Least Squares (PLS)/SVR/Extreme Learning Machine (ELM) modeling	R^2^ = 0.984	[104]
Wheat	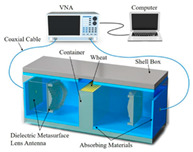	Dielectric Hypersurface Lens Antenna; density-independent calibration	RMSE = 0.178%	[132]
Peanut-hull pellets and pine-sawdust pellets	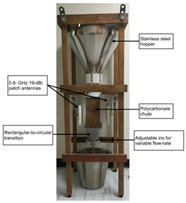	Single-frequency (5.8 GHz) density-independent calibration via permittivity function	Moisture Root mean square deviation (RMSD) < 0.5% (flowing vs. static) at 0.2–3.2 kg/s flow rates	[133]
Rice and maize	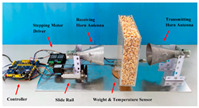	Traveling–standing wave attenuation	Rice: R^2^ = 0.988, SEP = 0.59% Maize: R^2^ = 0.991, SEP = 0.34%	[133]
Wheat and soybean	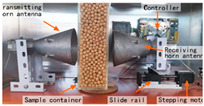	Traveling–standing wave nodes; density-independent calibration	Soybean: R^2^ = 0.995, SEP = 0.56% Wheat: R^2^ = 0.975, SEP = 0.98%	[73]
Single in-shell peanuts	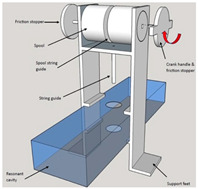	Resonant cavity perturbation at 3.7–4.1 GHz (measuring Δf, Q factor, S21); dielectric property calculation	AI classification accuracy: 86.3% (89.5% training) Intact/Empty ID accuracy: 91%/100%	[143]
Peanut (shelled)	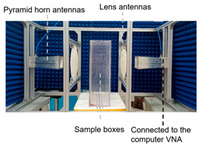	Scattering parameters (S-parameters); Fully Connected Deep Neural Network (FC-DNN) modeling	SEP = 0.56%	[136]
Peanut	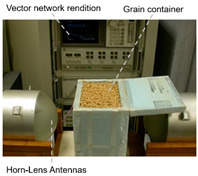	Dielectric method (pellets)	R^2^ = 1.000, RMSE = 0.159%	[92]
Unshelled tea seeds	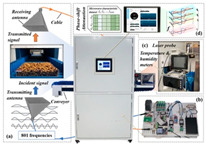	Multi-frequency (2–10 GHz) microwave scanning (attenuation and phase shift spectra) with ANN modeling and moisture calibration function	ANN R^2^ = 0.988–0.996 for seed MC; calibrated kernel MC R^2^ = 0.989; and MC range: 6.94–26.88% wet basis	[138]
Tea leaves	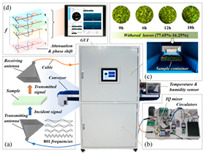	Multi-frequency optimization (Ant Colony Optimization), stacked ensemble modeling	R^2^ = 0.994	[139]
Oil–water mixtures	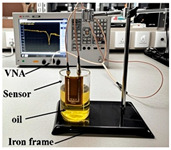	Nested-Complementary Split Ring Resonator (NCSRR); resonance frequency shift	An increase in the dielectric constant of the liquid to be measured leads to a linear decrease in the resonant frequency of the sensor	[105]
Tomato and tobacco	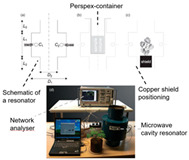	Microwave resonator; dielectric shift vs. fresh weight	Tomato: R^2^ = 0.93 Tobacco: R^2^ = 0.81	[100]
Plant leaves	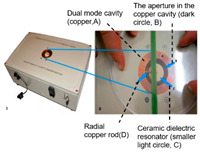	Dual-frequency resonator (2.4 GHz/150 MHz); water content and ionic conductivity	Correlation (*p* < 0.05)	[141]
Silage	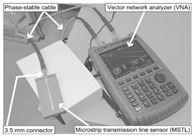	Microstrip transmission line; phase-amplitude ratio (Δφ/ΔA)	Barley: R^2^ = 0.962 Maize: R^2^ = 0.808	[142]
